# Development of a Hybrid Bioinorganic Nanobiocatalyst: Remarkable Impact of the Immobilization Conditions on Activity and Stability of β-Galactosidase

**DOI:** 10.3390/molecules26144152

**Published:** 2021-07-08

**Authors:** Luigi Tavernini, Oscar Romero, Carla Aburto, Fernando López-Gallego, Andrés Illanes, Lorena Wilson

**Affiliations:** 1 Escuela de Ingeniería Bioquímica, Pontificia Universidad Católica de Valparaíso, Avenida Brasil 2085, Valparaíso 2362803, Chile; Luigi.tavernini@pucv.cl (L.T.); carla.aburto@pucv.cl (C.A.); andres.illanes@pucv.cl (A.I.); 2 Bioprocess Engineering and Applied Biocatalysis Group, Department of Chemical Biological and Environmental Engineering, Universitat Autònoma de Barcelona, 08193 Bellaterra, Spain; 3 CIC biomaGUNE, Basque Research and Technology Alliance (BRTA), Paseo de Miramón 182, 20014 Donostia-San Sebastián, Spain; flopez.ikerbasque@cicbiomagune.es; 4 Ikerbasque, Basque Foundation for Science, 48013 Bilbao, Spain

**Keywords:** β-galactosidase, nanoimmobilization, biomineralization

## Abstract

Hybrid bioinorganic biocatalysts have received much attention due to their simple synthesis, high efficiency, and structural features that favor enzyme activity and stability. The present work introduces a biomineralization strategy for the formation of hybrid nanocrystals from β-galactosidase. The effects of the immobilization conditions were studied, identifying the important effect of metal ions and pH on the immobilization yield and the recovered activity. For a deeper understanding of the biomineralization process, an in silico study was carried out to identify the ion binding sites at the different conditions. The selected β-galactosidase nanocrystals showed high specific activity (35,000 IU/g biocatalyst) and remarkable thermal stability with a half-life 11 times higher than the soluble enzyme. The nanobiocatalyst was successfully tested for the synthesis of galacto-oligosaccharides, achieving an outstanding performance, showing no signs of diffusional limitations. Thus, a new, simple, biocompatible and inexpensive nanobiocatalyst was produced with high enzyme recovery (82%), exhibiting high specific activity and high stability, with promising industrial applications.

## 1. Introduction

The need to improve the economic and catalytic performance of enzymes for industrial application has driven the development of numerous immobilization strategies aimed at enhancing biocatalyst stability (either thermal or mechanical) to re-use the enzymes in multiple operational cycles [1]. In addition, recent advances in enzyme immobilization not only allow their reuse, but also improve and modulate their catalytic properties [2,3]. Nevertheless, immobilization often impacts negatively on enzyme activity by causing inactivation due to the harsh immobilization conditions, and by adding inert mass in the form of a carrier [4]. Consequently, much effort has been spent in optimizing the immobilization protocols to reduce these negative effects and making the advantages of immobilization prevail. In this direction, during the last decades there has been growing interest in the synthesis and use of nanostructured materials for enzyme immobilization. Nanostructured materials display a large surface-to-volume ratio and highly adjustable surface characteristics, allowing an increased enzyme loading per unit mass, and immobilization conditions tailored for the specific requirements of the proteins [5]. The nanobiocatalysts thus developed exhibit improved immobilization efficiency, high stability, easy handling and recovery, as well as reduced mass transfer resistance for substrates, facilitating biotechnological applications [6]. In recent years, Ge et al. [7,8] introduced an innovative immobilization strategy based on the biomineralization of metal phosphates with proteins, resulting in what has been termed hybrid organic-inorganic nanoflowers, or simply nanoflowers, due to the flower-resembling nanostructure of these biocatalysts [9]. Hybrid nanoflowers have drawn the interest of researchers owing to the large surface reaction area offered by their petal-like nanostructures, increasing immobilization efficiency and substrate access, as well as stability and selectivity. Another outstanding characteristic is their very simple, eco-friendly, and cost-efficient synthesis [10,11,12]. The biomineralization process is carried out under mild reaction conditions at room temperature in aqueous phosphate buffer, harnessing the strong affinity of enzymes for metal ions which bind to the protein backbone via coordination interactions with reactive aminoacidic groups, forming nucleation points distributed across the protein surface [7,13,14].

Many factors have been reported to affect the formation and structure of the nanoflowers, including the concentration of reagents (enzymes, phosphate, and metal ions), pH, temperature, and biomineralization time [15]. However, to date little is known about how these factors affect the catalytic properties of the biocatalyst, such as expressed activity and stability [12,16]. Therefore, immobilization conditions must be thoroughly investigated to determine those yielding the optimal biocatalytic performance. This is crucial for enzymes for industrial use, where having a biocatalyst with good catalytic performance must be balanced with the economic viability of the process. This is the case of β-galactosidase (β-gal), a well-known enzyme with wide applications in the production of lactose-free products or prebiotics, such as galacto-oligosaccharide (GOS), lactulose and other syrups [17,18,19,20]. The need to improve the economic and catalytic performance of β-galactosidases for industrial application has driven the development of numerous immobilization strategies [21]. Despite the growing interest, the immobilization of β-galactosidase as bioinorganic nanoflowers has received little attention, and not much emphasis has been placed on the immobilization conditions, nor on its application in synthetic reactions [22,23,24].

The aim of this work was to evaluate the influence of the immobilization condition on the catalytic properties of the nanobiocatalyst, focusing on the effects of metal ions, salt/protein ratio, biomineralization time and pH. Furthermore, in order to get insight into the biomineralization process, a computational study was carried out to identify the potential nucleation sites of the enzyme at different immobilization conditions. Finally, the nanobiocatalyst was assessed in terms of its expressed activity, thermostability and its performance in the synthesis of GOS.

## 2. Results and Discussion

### 2.1. Metal Ion Screening

A screening was conducted with Cu^2+^, Mg^2+^, Zn^2+^, Co^2+^, Fe^2+^, Ca^2+^ to determine those ions suitable for the immobilization of β-gal as hybrid bioinorganic nanocrystals. The results in terms of immobilization yield (IY) led to the selection of Ca^2+^ for the following research stages, given the substantial advantage shown by this ion over the other ions assayed, obtaining an IY of 29.4%, which exceeds by five times the IY of the next best ion (Cu^2+^) (Table S1, Supplementary Materials).

### 2.2. Biomineralization of β-Galactosidase with Calcium

**Figure 1:**
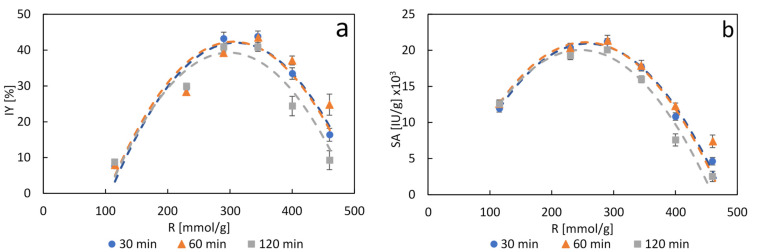
Immobilization yield (IY) (**a**) and specific activity (SA) (**b**) of calcium-phosphate nanocrystals of β-galactosidase produced at pH 7.4 at CaCl_2_/protein ratios (R) of 115, 230, 290, 345, 400 and 460 mmol_CaCl_2__/g_protein_ for 30 min, 60 min, and 120 min of biomineralization time. Data are presented as mean ± margin of error, *n* = 2. Dashed lines represent the model for each data set according to Equation (S1} (Supplementary Materials).

Once the calcium ion was selected, the study of biomineralization at pH 7.4 was conducted using CaCl_2_/protein ratios of 115, 230, 290, 345, 400 and 460 mmol_CaCl_2__/g_protein_, and contact times of 30, 60 and 120 min. The results obtained in terms of immobilization yield (IY) and specific activity (SA) (Figure 1) showed an optimum in the range from 290 to 345 mmol_CaCl_2__/g_protein_ in the case of IY, and around 290 mmol_CaCl_2__/g_protein_ in the case of SA, regardless of the biomineralization time evaluated. Maximum IY was 44% at 345 mmol_CaCl_2__/g_protein_, which is very close to the value of 43% obtained at 290 mmol_CaCl_2__/g_protein_ with a negligible difference between 30 and 60 min of biomineralization. A peak of 21,300 IU/g_biocatalyst_ was achieved unambiguously at 290 mmol_CaCl_2__/g_protein_, again showing no significant difference between 30 and 60 min of biomineralization. Overall IY and SA slightly decreased at a biomineralization time of 120 min, in comparison to 30 and 60 min.

Remarkably, IY and SA followed the same trend, and hence exhibited the same optimum, unlike most other immobilization methods, particularly the support-based ones, in which enzyme load per unit mass of carrier usually results in a clear compromise between IY and SA. A very close trend to the one shown in Figure 1 can be observed for protein immobilization (Figure S1, Supplementary Materials) being the optimum protein immobilization yield obtained between 290 and 345 mmol_CaCl_2__/g_protein_ (62% in nanocrystals produced after 60 min of biomineralization, and 50% in nanocrystals produced after both 30 and 120 min of biomineralization). Diffusional limitations may be playing a role on the activity drop in the CaCl_2_/protein range beyond the optimum (around 290 mmol_CaCl_2__/g_protein_), although, as seen in Figure S1, protein immobilization followed a similar trend to the SA and IY in Figure 1, showing that at higher CaCl_2_/protein ratios less protein was being biomineralized. This is possibly due to the formation of more naked calcium-phosphate salt at the expense of the protein-calcium-phosphate complex. On the other hand, the detrimental effect observed at 120 min of biomineralization may be caused by modifications of the enzyme’s structure leading to activity loss, or due to increasing diffusional restrictions brought about by more compact and complex structures hindering substrate access. It has been widely reported that the structure complexity of nanoflowers increase with incubation time [11,15].

The results were arranged into a full 32 factorial experimental design to simplify analysis and subjected to an analysis of variance (ANOVA) to assess the statistical significance of the variables CaCl_2_/protein ratio and biomineralization time (see Supplementary Materials). Considering a level of significance of α = 0.05, both variables, as well as their combination, exerted a statistically significant effect on IY and SA of the β-gal nanocrystals, validating the previous observations drawn from Figure 1.

### 2.3. Effect of pH on the Biomineralization of β-Galactosidase

Enzyme immobilization as hybrid bioinorganic nanoflowers has been shown to be critically affected by pH. Despite this, few examples of biomineralization at different pHs are described in the literature [15], motivating the present study of the effect of pH on biomineralization.

**Figure 2:**
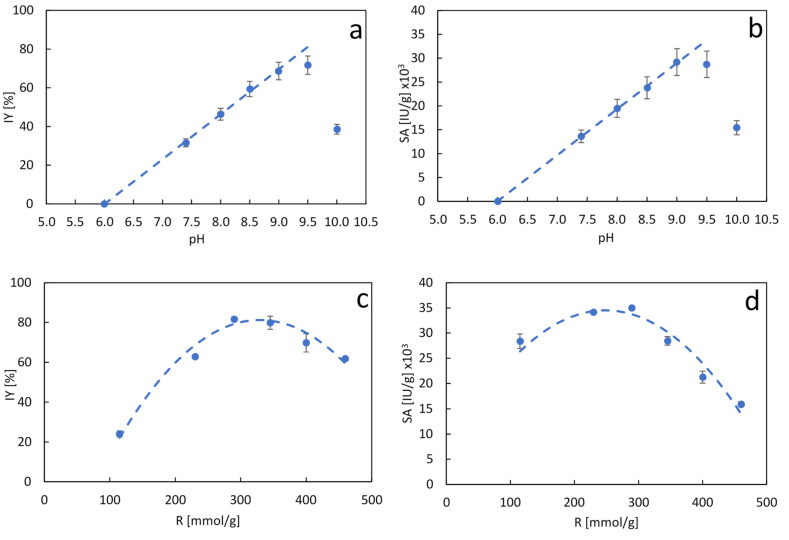
Immobilization yield (IY) (**a**) and specific activity (SA) (**b**) of calcium-phosphate β-galactosidase nanocrystals produced at a CaCl_2_/protein ratio of 290 mmol_CaCl_2__/g_protein_ for 60 min at different pHs. Immobilization yield (IY) (**c**) and specific activity (SA) (**d**) of calcium-phosphate β-galactosidase nanocrystals produced at pH 9.0 for 60 min at different CaCl_2_/protein ratios. Data are presented as mean ± margin of error, *n* = 2. Dashed lines in (**c**) and (**d**) represent the model for each data set according to Equation (S2) (Supplementary Materials).

The effect of pH on β-gal biomineralization was firstly evaluated by means of prospective assays carried out at 290 mmol_CaCl_2__/g_protein_ for 60 min. The results show a linear correlation in the pH range from 6 to 9 for both IY and SA (Figure 2a,b). Beyond pH 9.0, a sudden drop in both parameters was observed, while at pH below 6.0 no activity was recovered. Quite remarkably, IY increases from 0 at pH 6.0 to 72% at pH 9.0, demonstrating the strong effect of pH on the biomineralization process.

The effect of pH on the formation of nanoflowers is presumably due to changes in the ionization state of the enzyme’s reactive groups as well as to changes in the distribution equilibrium of the phosphate species [13,25]. Several authors have suggested that an increase of the net negative charge of the enzyme molecules at higher pH had a positive effect on the binding affinity of metal ions, promoting biomineralization by increasing nucleation points [26]. Furthermore, previous works have reported that an increase in pH yielded less ordered and less compact nanoflower structures [26,27]. This could favor the diffusion of substrates and products in and out from the biocatalyst, explaining the increase in specific activity at higher pHs.

These results led to the formulation of a new experimental run at pH 9.0, considering the same CaCl_2_/protein ratio range assayed at pH 7.4 but considering only 60 min of biomineralization. As control, we incubated the soluble enzyme at the biomineralization pH in order to detect potential activity loss. As a result, we observed that the enzyme remained fully active for up to 3 h of incubation in PBS at pH 9. Figure 2c,d displays the behavior of IY and SA in relation to the CaCl_2_/protein ratio as a result of the biomineralization of β-gal with calcium-PBS at pH 9.0 for 60 min. As was the case for the biomineralization at pH 7.4, production of the β-gal nanocrystals at pH 9.0 exhibited a quadratic correlation of both IY and SA with respect to the CaCl_2_/protein ratio, with a clear maximum in the vicinity of 290 mmol_CaCl_2__/g protein. The maximum values of SA and IY were 35,000 IU/g_biocatalyst_ and 81.7% respectively, showing a remarkable improvement over previous results at pH 7.4, confirming the successful optimization in the biocatalyst preparation. These data were modeled according to the quadratic expression (model and parameters are shown in Supplementary Information). ANOVA testing showed that the regression model is highly significant, supporting the critical effect of both pH and CaCl_2_/protein ratio on the biomineralization of β-gal.

### 2.4. Thermal Stability of the β-Galactosidase Nanocrystals

**Figure 3:**
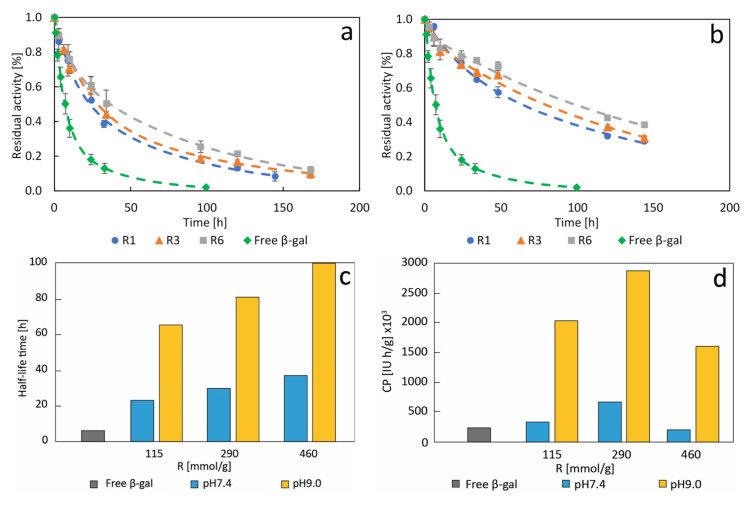
Inactivation kinetics at 50 °C and pH 7.0 of free β-galactosidase (β-gal) and β-galactosidase nanocrystals produced at pH 7.4 (**a**), and at pH 9.0 (**b**) after 60 min of mineralization time at CaCl_2_/protein ratios of 115 mmol_CaCl_2__/g_protein_ (R1), 290 mmol_CaCl_2__/g_protein_ (R3), and 460 mmol_CaCl_2__/g_protein_ (R6). Data are presented as mean ± margin of error, *n* = 2. The dashed lines represent the models. Half-life time (**c**) and catalytic potential (CP) (**d**) of free β-galactosidase (β-gal) and β-galactosidase nanocrystals produced after 60 min of mineralization time at pH 7.4 (pH 7.4, blue column) and pH 9.0 (pH 9.0, yellow column) at different CaCl_2_/protein ratios (R). The catalytic potential was evaluated at 30% residual activity.

The thermal stability of selected β-galactosidase nanocrystals was evaluated under nonreactive conditions 100 mM PBS pH 7.0 at 50 °C. Thermal stability of free β-gal was also carried out for comparative purposes. To do so, the stability tests were performed for the biocatalysts made at pH 7.4 and 9.0, considering 115, 290, and 460 mmol_CaCl_2__/g_protein_, as these CaCl_2_/protein ratios. As can be seen from Figure 3a,b, nanocrystals in general showed higher stability than soluble β-gal, the latter being almost completely inactivated after 100 h of incubation, while the β-gal nanocrystals made at pH 7.4 and pH 9.0 retained over 20% and 40% of activity, respectively, after the same incubation time. Another relevant finding derived from the inactivation kinetics of the β-gal nanocrystals was that stability increased proportionally to the CaCl_2_/protein ratio. This could be explained by the fact that an increase in CaCl_2_/protein ratio generates more compact and complex structures (Figure 3), creating a protective effect on the enzyme structure and the activity thereof. Parameters of the mathematical models for the inactivation kinetics are shown in Sup. Information. The ANOVA and coefficients of determination prove the statistical significance of the models, and the parameters half-life time, SF and CP were determined therefrom.

Figure 3c,d show the half-life times and the catalytic potential of different β-gal bioinorganic nanocrystals compared to the free enzyme. β-gal nanocrystals synthesized at pH 9.0 exhibit overall higher half-life times than the soluble enzyme and the nanocrystals produced at pH 7.4, respectively. A SF of 14.7 was achieved at the most stable condition (pH 9.0, 460 mmol_CaCl_2__/g_protein_ and 60 min of biomineralization), and the best immobilization condition showed a SF of 11.6 (pH 9.0, 290 mmol_CaCl_2__/g_protein_, 60 min). These results are substantially better than those reported with other immobilization methods, in Table 1 a comparison of the results of different methods of immobilization for *A. oryzae* β-galactosidase are shown. The developed biocatalyst exhibited both high SA and high stability as opposed to other works reviewed that usually yield a trade-off between these two parameters (Table 1).

The catalytic potential (CP) denotes the total catalytic capacity of the biocatalyst along its lifespan and allows the merging of activity of the immobilized biocatalysts and their stability into one compounded parameter [37]. Figure 3d shows the CP of different biocatalysts made under different conditions and compares them with the free enzyme. As shown in all previous results, the β-gal nanocrystals produced at pH 9.0 performed markedly better than those made at pH 7.4. The highest CP was obtained at 290 mmol_CaCl_2__/g_protein_ with a value of 2.868 × 10^3^ IU∙h/g_biocatalyst_, as compared to the 245.3 × 10^3^ IU∙h/g_biocatalyst_ of the free enzyme, highlighting the importance of optimization of the immobilization conditions (see Figure 3d).

### 2.5. In Silico Prediction of Metal-Ion Binding Regions

**Figure 4:**
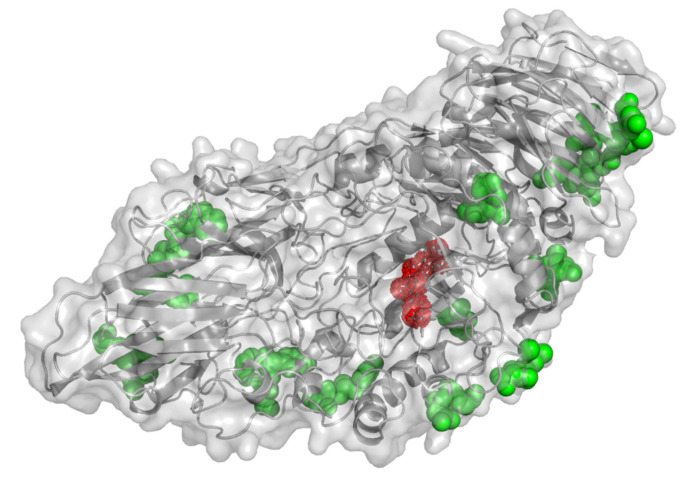
Predicted nucleation sites of calcium ions on β-galactosidase using MIB server (green) and active site of the enzyme (red).

In order to get insight into the biomineralization process, potential nucleation sites for Ca^2+^ were identified based on the structural information of β-gal (PDB ID: 4IUG) and a server for predicting metal ion binding sites (MIB at http://bioinfo.cmu.edu.tw/MIB/ (accessed on 11 June 2020)) [38]. Based on this methodology, potential sites of nucleation have been previously determined for different bioinorganic composites [13,39]. A great number of Ca^2+^ nucleation sites were predicted for β-gal, which may explain the fast and efficient formation of the bioinorganic composites. The predicted binding sites for β-gal are shown in Figure 4 and the Table S2 (Supplementary Materials), and as expected many of them involve amino acids usually found at calcium binding sites such as: Asp, Glu and Asn. It is also observed that no binding sites are located near the catalytic pocket, which supports the high recovered enzyme activity upon the biomineralization process.

Then, we were interested in understanding the effect of pH over the predicted nucleation points. To that aim, we determined the protonation states of each residue of β-gal at both pH 7.4 and 9.0 using the PlayMolecule repository [40]. The same nucleation sites were identified at both pHs, with no significant difference in their binding scores (Table S2, Supplementary Materials). This is probably because none of the amino acids involved in calcium binding undergo changes in their protonation state between pH 7.4 and 9.0 [38,40], suggesting that the difference in biomineralization at varying pHs may rather be due to changes in the distribution equilibrium of phosphate species (H_2_PO_4_^−^; HPO_4_^2−^).

Crystallization and precipitation of calcium phosphates is a complex phenomenon driven by both thermodynamic and kinetic interactions and affected by variables such as composition, supersaturation, temperature, pH, ionic strength and stoichiometric ratios [41].

The effect of pH is prevalent on the solution speciation and the net charge of the mineral surface. In alkaline solutions, the mineral surface is negatively charged and ions, such as H^+^ and Ca^2+^, bind to the surface forming complex structures [41]. Calcium phosphate speciation has been determined to involve no less than eleven variants, the most prominent of which are monocalcium phosphate, dicalcium phosphate, and apatites including octacalcium phosphate, amorphous calcium phosphate, hydroxyapatite and calcium deficient apatite [42]. Interactions with 3D-folded biomacromolecules such as β-gal add more complexity to the phenomenon, and thorough research must be conducted to elucidate the mechanisms involved, a task beyond the scope of this work.

### 2.6. Characterization of the β-Galactosidase Nanocrystals

**Figure 5:**
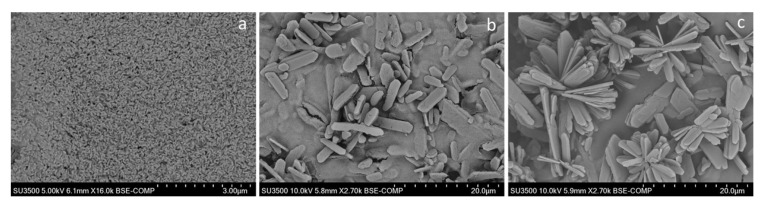
SEM photography of the calcium-phosphate β-galactosidase nanocrystals produced at pH 9.0 for 60 min at CaCl_2_/protein ratios of 115 mmol_CaCl_2__/g_protein_ (**a**), 290 mmol_CaCl_2__/g_protein_ (**b**), and 460 mmol_CaCl_2__/g_protein_ (**c**).

SEM imagery was taken for samples of the β-gal nanocrystals made at pH 9.0 for 60 min and at different CaCl_2_/protein ratios (Figure 5). Most studies describe a three-step process: in a first stage, metal ions bind to proteins via coordination groups forming nucleation points for the metal-phosphate crystals; in a second growth stage, the protein-metal-phosphate complexes aggregate into larger petal-like agglomerates, which finally develop into full multilayered flower-like structures in a third stage of anisotropic growth [9,43]. Many studies have looked into the kinetics of the process, confirming the presence of irregular nanocrystals at very early stages (0.1 h to 2 h), followed by flower-mimicking nanostructures with increasing incubation time (3 h to 24 h), yielding compact nanoflowers after about 24 h, with the time-frames varying in each particular case [44,45]. Figure 5 shows a development process as a function of the CaCl_2_/protein ratio. As in the kinetics of the process, Figure 5a–c shows the effect of calcium/protein ratio on the morphology of the bioinorganic composites. While a ratio of 115 mmol_CaCl_2__/g_protein_ forms nanoparticles, a much higher ratio (460 mmol_CaCl_2__/g_protein_) forms flower-like structures. Rectangular microcrystal of approximately 2 × 10 µm were found at intermediate ratio, indicating that the CaCl_2_/protein clearly influences the hierarchical assembly of bioinorganic nanocrystals. Other structures, beside nanoflowers, have been reported for calcium-based nanocrystals as those found in this work [46]. The stability tests in Figure 3 suggest that the higher structural complexity associated with higher CaCl_2_/protein ratios also plays a role in enhancing stability, possibly due to bigger and more robust structures exerting a protective effect on the enzyme.

### 2.7. Synthesis of Galacto-Oligosaccharides

**Figure 6:**
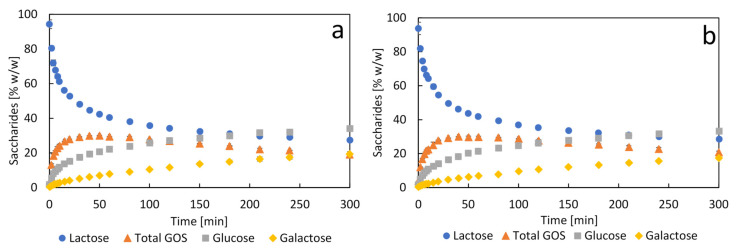
Kinetics of synthesis of galacto-oligosaccharides with free (**a**) and bioinorganic nanocrystals (**b**) of β-galactosidase. Synthesis conditions: 100 IU/g_lactose_, lactose 50% *w*/*w* in citrate-phosphate buffer pH 4.5 and 50 °C. Data are presented as mean ± margin of error, *n* = 2.

The bioinorganic β-gal nanocrystals were tested as catalysts in the synthesis of GOS. The results in terms of percentage of total saccharides can be seen in Figure 6. GOS of varying length, namely GOS-3, GOS-4 and GOS-5 were considered as total GOS. In Figure 6, the profiles of the hydrolysis products glucose and galactose are also represented, and the remaining lactose. The kinetics of GOS synthesis with free β-gal (Figure 6a) and with β-gal nanocrystals (Figure 6b) mirror each other almost exactly, both reaching a maximum GOS yield (30%) at about 40 min. These results agreed with those obtained by Vera et al. (2012) at the same conditions using the same soluble β-gal from *A. oryzae*. GOS conversion yields obtained with β-gal from *A. oryzae* using other immobilization methods usually range from 15 to 27% [47], which highlights the good performance of the biocatalysts herein presented. These are remarkable results since immobilized enzymes usually show poorer performance than the free enzymes due to diffusional limitations [48]. This supports the notion that nanoflowers, or nanocrystals, are not subjected to significant mass transfer limitations due to their large reactive area, suggesting that the vast majority of the immobilized enzymes are highly exposed to the bulk of the reaction. The lower performance of immobilized biocatalysts in relation to the soluble enzymes is a feature that reduces productivity, therefore compromising the competitiveness of these biocatalysts and sets out serious concerns about their potential use as catalysts for industrial processes.

The β-gal nanocrystals showed no signs of diffusional limitations and no negative impact on productivity. Furthermore, considering the high catalytic potential of the β-gal nanocrystals, as reflected on a SF value of 11.6, it is safe to conclude that the biocatalyst developed has promising prospects for GOS synthesis, and possibly for lactulose synthesis and lactose hydrolysis, as well. Additionally, the biocompatibility of calcium opens up multiple applications for this type of biocatalyst in the areas of functional foods and biomedicine [49,50].

## 3. Materials and Methods

### 3.1. Chemicals and Reagents

Commercial β-galactosidase Enzeco Fungal Lactase from A. oryzae (β-gal) (EC 3.2.1.23) with a specific activity (SA) of 152,361 ± 4755 IU/g_protein_ was supplied by Enzyme Development Corporation, EDC (New York, NY, USA). All chemicals were purchased from Sigma Aldrich (St. Louis, MO, USA) at the highest purity available.

### 3.2. Hydrolytic Activity of β-Galactosidase

One international unit of hydrolytic activity of β-galactosidase (IU) was defined as the amount of enzyme releasing 1 µmol of o-NP per min as a result of the hydrolysis of o-NPG at pH 7.0 and 40 °C. Molar extinction coefficient of o-NP was 2.21 mM^−1^cm^−1^ at 420 nm.

### 3.3. Biomineralization of β-Galactosidase

The biomineralization procedure was based on the method reported by Escobar et al. [13] where 400 μL of a metal salt solution (CuSO_4_, MgSO_4_, ZnCl_2_, CoCl_2_, FeSO_4_, CaCl_2_) were added under magnetic stirring to 5 mL of a 1 mg/mL enzyme solution in phosphate-buffered saline (PBS) made from 50 mM sodium phosphate and 150 mM NaCl at room temperature. Biomineralization time (min, t), pH of the PBS solution, and metal/protein ratio (mmol of metal salt/g of contacted protein, R) were established as required by the experimental conditions under study (details in Supplementary Materials). The resulting biomineral suspension was subjected to centrifugation at 10,000 rpm for 15 min and then rinsed and resuspended in 5 mL of 25 mM potassium phosphate buffer pH 7.0, repeating the process two further times. The β-gal nanocrystals were then stored at 4 °C in rinsing buffer.

All results were evaluated in terms of specific activity (SA) and immobilization yield (IY) both defined according to:

(1) $$SA=A_{E}/M_{b}\cdot 100$$

(2) $$IY=A_{E}/A_{I}\cdot 100$$

where *A_E_* is the total activity expressed by the enzyme or immobilized biocatalyst (IU), *M_b_* is the dry weight of the biocatalyst (g), and *A_I_* is the initial activity subjected to immobilization (IU).

SA was expressed in international units of hydrolytic activity per g of protein in the case of free β-gal (IU/g_protein_), and in international units of hydrolytic activity per g of biocatalyst in the case of the β-gal bioinorganic nanocrystals (IU/g_biocatalyst_).

### 3.4. Thermal Stability of the β-Galactosidase Bioinorganic Nanocrystals

A selection of the bioinorganic β-gal nanocrystals developed and free β-gal were subjected to thermal inactivation at 50 °C in 100 mM PBS pH 7.0. The resulting kinetics of inactivation were modeled according to a series biphasic inactivation mechanism without residual activity [37]. The model equations are described in Supporting Information. Additionally, half-life time (t_1/2_), stabilization factor (SF) and catalytic potential (CP) were determined for the β-gal nanocrystals from the thermal inactivation kinetics. The description and equations for these parameters is presented in Supplementary Materials.

### 3.5. In Silico Prediction of Calcium Binding Sites

The possible sites of nucleation of β-gal (PDB: 4IUG) were determined using the fragment transformation method available as a public server at MIB (http://bioinfo.cmu.edu.tw/MIB/introduction (accessed on 11 June 2020)) [38]. The protonation states of each residue at pH 7.4 and 9.0 was determined using the ProteinPrepare at PlayMolecule repository [40]. Both protonation states were evaluated in MIB server for the prediction of metal binding sites for Ca^2+^.

### 3.6. Characterization of the β-Galactosidase Bioinorganic Nanocrystals

The calcium-phosphate β-gal nanocrystals were analyzed using scanning electron microscopy (SEM) using a HITACHI SU 3500 instrument. The samples were previously washed with distilled water to remove unspecific crystals from the phosphate buffer and then sputtered with gold to minimize surface charging.

### 3.7. Synthesis of Galacto-Oligosaccharides

The developed immobilized biocatalyst was tested in the synthesis of GOS along with the free β-gal for comparative purposes. The procedure and reaction conditions were based on the work by Vera et al. [51]. The reaction assays were conducted in duplicates in 150 mL Erlenmeyer flasks at 50 °C under magnetic stirring. Lactose monohydrate was added at 50% *w*/*w* (12.5 g) in 100 mM citrate-phosphate buffer pH 4.5 dissolving it by heating at a temperature close to 100 °C. The reaction was initiated once the free β-gal solution or β-gal biocatalyst was added at a ratio of 100 IU/g_lactose_. The products of the reactions were analyzed by HPLC in a Jasco RI 2031 delivery system coupled with a refractive index detector using a BP-100 Ca^2+^ column (300 mm × 7.8 mm) (Benson Polymeric, Reno, NV, USA) and milli-Q water as mobile phase [51].

## 4. Conclusions

This study reports the successful immobilization of *A. oryzae* β-galactosidase as hybrid bioinorganic nanocrystals. Calcium was determined to be the most adequate metal ion for β-gal biomineralization, outperforming copper by five times in terms of immobilization yield, whereas almost negligible results were obtained with the other ions tested. Conditions such as metal/protein ratio and pH proved to be highly relevant. CaCl_2_/protein ratio showed an optimum of both immobilization yield and specific activity in the vicinity of 290 mmol_CaCl_2__/g_protein_. The metal/protein ratio was also influential on the hierarchical assembling of the nanocrystals with larger, more complex and more robust structures at higher metal/protein ratios, as confirmed by SEM and the stability tests. Particularly significant was the effect of pH, allowing an increase in the immobilization yield from 0 to 82% in the pH range from 6.0 to 9.0, clearly demonstrating the high importance of optimization of the immobilization conditions in the development of this types of nanobiocatalyst. The in silico analysis showed no correlation between the predicted nucleation points and shifts in the ionization state of critical reactive groups as a consequence of pH change. Although such correlation cannot be completely dismissed, the effect of pH might be rather related to variations of calcium phosphate speciation.

The developed biocatalyst showed a specific activity of 35,000 IU/g_biocatalyst_, and a stability factor of 11.6 (at 50 °C), surpassing the results reported to date for other immobilized β-gal in which a compromise usually occurs between both parameters. Furthermore, the bioinorganic β-gal nanocrystals were successfully tested for the synthesis of GOS performing as well as the free enzymes in terms of yield and volumetric productivity, showing therefore, no signs of diffusional limitations. These features make the β-gal nanocrystals highly competitive biocatalysts for the synthesis of GOS and for other important industrial and biomedical applications.

In short, β-galactosidase immobilization as bioinorganic nanocrystals was shown to be a remarkably simple cost-efficient methodology that allows high retention of enzyme activity, producing a biocatalyst with high specific activity, high stability, and optimal performance of GOS synthesis from lactose. Furthermore, because calcium is safe and compatible as a food and pharmaceutical additive, multiple applications open up for this biocatalyst in the production of prebiotics and drug-delivery.

## Figures and Tables

**Table 1:** Comparative results of immobilization of *Aspergillus oryzae* β-galactosidase by different methods and thermal stability of the resulting biocatalysts.

Immobilization Methods	Support/Precipitant	Immobilization Yield [%]	Specific Activity [IU/g] ^1^	Stability Factor	Reference
Biomineralization	-	81.7	35,000	11.6	This work
Covalent bonding	Glyoxyl-agarose	85	2500	1.5	[28]
	Amino-glyoxyl-agarose	57	7700	2.3	[28]
	Carboxy-glyoxyl-agarose	13.14	780	1.3	[28]
	Chelate-glyoxyl-agarose	5.13	455	1.4	[28]
	Chitosan-epichlorohydrin	54	2951	1.8	[29]
	Amino-glyoxyl-agarose	40	2294	2.5	[29]
	Chitosan-glutaraldehyde	70.5	3070	3.9	[30]
	Alginate-cellulose beads	70	n.r.	3.4	[31]
	Chitosan-glutaraldehyde	18.4	n.r.	1.2	[32]
	Cotton fibers-PEI	80	n.r.	10.3	[33]
	Sodium alginate	n.r.	n.r.	2.8	[34]
CLEAs	-	100	64,000	1.1	[35]
	-	90	44,560	2.3	[35]
	-	13.5	Not reported	1.2	[32]
	-	30	15,000	1.3	[36]

^1^ Activity determined using o-NPG as substrate. n.r.: not reported.

## Data Availability

Not applicable.

## Supplementary Materials

Figure S1. Protein immobilization yield (IY_P_) of calcium-phosphate nanocrystals of β-galactosidase produced at pH 7.4 for biomineralization times of 30 min (T30), 60 min (T60), and 120 min (T120), and salt/protein ratios (R) of 115, 230, 290, 345, 400 and 460 mmol_CaCl_2__/g_protein_; Table S1. Immobilization yield of β-galactosidase bioinorganic nanocrystals using several metal ions. Biomineralization conditions: PBS pH 7.4, 230 mmol_salt_/g_protein_, for 2 h at room temperature; Table S2. Binding residues and scores predicted for Ca^+2^ by the MIB server; Table S3. Results of the 3^2^ full factorial experimental design for the biomineralization of β-galacto-sidase with calcium-phosphate. Variables: time (t), and salt/protein ratio (R). Response parame-ters: specific activity (SA) and immobilization yield (IY); Table S4. ANOVA test of the 3^2^ full factorial experimental design for the biomineralization of β-galactosidase with calcium-phosphate. Variables: time (t), and salt/protein ratio (R). Response pa-rameter: specific activity (SA); Table S5. ANOVA test of the 3^2^ full factorial experimental design for the biomineralization of β-galactosidase with calcium-phosphate. Variables: time (t), and salt/protein ratio (R). Response pa-rameter: immobilization yield (IY); Table S6. Parameters of the models developed for the immobilization yield (IY) and specific activ-ity (SA) of the biomineralization of β-galactosidase with calcium-phosphate according to Equation (S4); Table S7. Parameters, *p*-values, and coefficients of determination (R^2^) of the models developed for the immobilization yield (IY) and specific activity (SA) resulting from the biomineralization of β-galactosidase at pH 9.0 for 60 min. The models are represented by Equation (S5); Table S8. Model parameters, *p*-values, and coefficients of determination of the kinetic models of inactivation of β-galactosidase nanocrystals produced at pH 7.4 and pH 9.0 for 60 min of bio-mineralization at calcium/protein ratios of 115 mmol_CaCl_2__/g_protein_ (R1), 290 mmol_CaCl_2__/g_protein_ (R3), and 460 mmol_CaCl_2__/g_protein_ (R6). The models correspond to the biphasic inactivation mechanism as represented by Equation (S6). Equation (S1): Multiple linear regression; Equation (S2): Regression models for the experimental designs.

## Sample Availability

Samples of the compounds are not available from the authors.
