# Supplementary material for: Development of a Hybrid Bioinorganic Nanobiocatalyst: Remarkable Impact of the Immobilization Conditions on Activity and Stability of β-Galactosidase
Source: Molecules. 2021 Jul 8;26(14):4152. doi: 10.3390/molecules26144152 (PMC8303607; doi:10.3390/molecules26144152)
Supplement: Supplementary file 1 [file molecules-26-04152-s001.zip › molecules-1271631-supplementary.pdf]

Supplementary Material

Development of a Hybrid Bioinorganic Nanobiocatalyst: Remarkable Impact of the Immobilization Conditions on Activity and Stability of β-galactosidase

Luigi Tavernini[1], Oscar Romero [1,2,*], Carla Aburto [1], Fernando López-Gallego [3,4], Andrés Illanes [1], Lorena Wilson [1,*]

[1] Escuela de Ingeniería Bioquímica, Pontificia Universidad Católica de Valparaíso, Avenida Brasil 2085, Valparaíso, 2362803, Chile
[2] Bioprocess Engineering and Applied Biocatalysis Group, Department of Chemical Biological and Environmental Engineering, Universitat Autònoma de Barcelona, 08193, Bellaterra, Spain
[3] CIC biomaGUNE, Basque Research and Technology Alliance (BRTA), Paseo de Miramón 182, 20014 Donostia – San Sebastián, Spain
[4] Ikerbasque, Basque Foundation for Science, 48013 Bilbao, Spain

Table S1. Immobilization yield of β-galactosidase bioinorganic nanocrystals using several metal ions. Biomineralization conditions: PBS pH 7.4, 230 $mmol_{salt}/g_{protein}$, for 2 h at room temperature.

**Metal ion**	**IY [%]**
Cu^{2+}	5.7
Mg^{2+}	0.0
Zn^{2+}	1.7
Co^{2+}	1.3
Fe^{2+}	0.2
Ca^{2+}	29.4

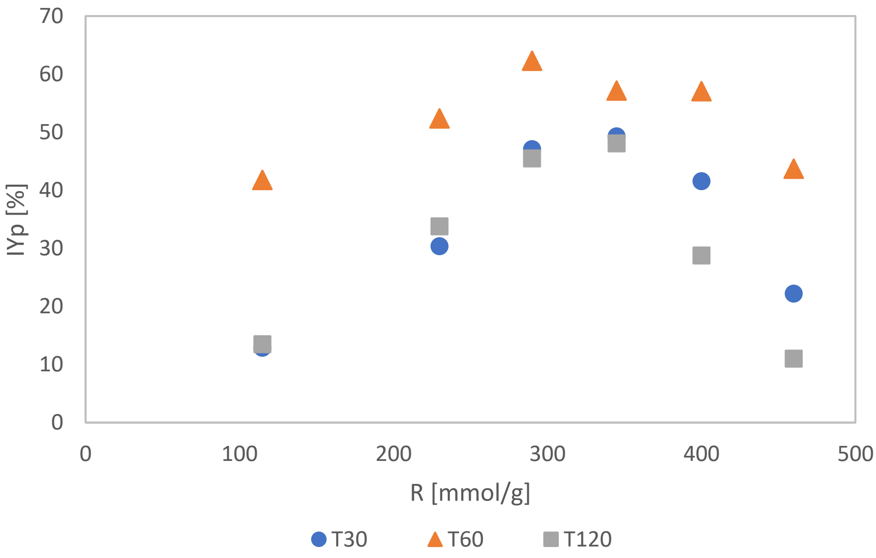

.

Figure S1. Protein immobilization yield (IY_P) of calcium-phosphate nanocrystals of β-galactosidase produced at pH 7.4 for biomineralization times of 30 min (T30), 60 min (T60), and 120 min (T120), and salt/protein ratios (R) of 115, 230, 290, 345, 400 and 460 $mmol_{CaCl2}/g_{protein}$

Table S2. Binding residues and scores predicted for Ca^{+2} at pH 7.4 and pH 9.0 by the MIB server.

	**Predicted binding site**	Score	
		pH 7.4	pH 9.0
**1**	689S , 690Y , 691D	3.578	3.580

2	844D，845Y，846Q	3.187	3.189
3	108D，109Y	3.130	3.128
4	214D，217Q	3.053	3.059
5	190G，191P	3.036	3.042
6	681D，682T，888G	2.828	2.828
7	476D，477Y，478N	2.565	2.570
8	676D，677W，678K	2.513	2.518
9	558G，559D	2.392	2.392
10	40D，41L	2.369	2.374
11	330N，331D	2.366	2.371
12	58E，59R	2.360	2.365
13	449T，453N	2.324	3.322

Table S3. Results of the 3^2 full factorial experimental design for the biomineralization of β-galactosidase with calcium-phosphate. Variables: time (t), and salt/protein ratio (R). Response parameters: specific activity (SA) and immobilization yield (IY).

**Codified Values**		**Real values**					
**t**	**R**	**t [min]**	**R [mmol/g]**	SA [IU/g] x10^3		IY [%]	
				A	B	A	B
0	0	30	115	11.59	12.33	7.52	8.01
1	0	60	115	12.41	12.73	7.85	8.06
2	0	120	115	12.19	12.98	8.50	9.05
0	1	30	290	21.83	20.57	44.51	41.94
1	1	60	290	21.54	21.14	39.59	38.85
2	1	120	290	20.32	19.84	41.44	40.46
0	2	30	460	4.27	5.00	15.08	17.66
1	2	60	460	6.76	8.02	22.64	26.88
2	2	120	460	2.01	3.06	7.35	11.18

Table S4. ANOVA test of the 3^2 full factorial experimental design for the biomineralization of β-galactosidase with calcium-phosphate. Variables: time (t), and salt/protein ratio (R). Response parameter: specific activity (SA).

**Factor**	**Sum of Squares**	**Degrees of Freedom**	**Mean Square**	F_0	***p*-value**
t	12.52	2	6.26	17.42	$7.66 \cdot 10^{-05}$
R	770.75	2	385.37	1072.45	$1.30 \cdot 10^{-18}$
tR	13.65	4	3.41	9.49	$3.20 \cdot 10^{-04}$
Error	3.23	9	0.36		
Total	800.15	17			

Table S5. ANOVA test of the 3^2 full factorial experimental design for the biomineralization of β-galactosidase with calcium-phosphate. Variables: time (t), and salt/protein ratio (R). Response parameter: immobilization yield (IY).

**Factor**	**Sum of Squares**	**Degrees of Freedom**	**Mean Square**	F_0	***p*-value**
t	57.49	2	28.75	10.81	$9.34 \cdot 10^{-04}$
R	3507.02	2	1753.51	659.52	$7.75 \cdot 10^{-17}$
tR	200.48	4	50.12	18.85	$4.47 \cdot 10^{-06}$
Error	23.93	9	2.66		
Total	3788.92	17			

Results from the experimental run at pH 7.4 were modeled according to a multiple linear regression as represented by the following expression:

$$y = \beta_0 + \beta_1 t + \beta_{12} R + \beta_{11} t^2 + \beta_{22} R^2 \qquad \text{Equation S1}$$

Where y could be SA or IY, β_0, β_1, β_{12} β_{11} and β_{22} are the model coefficients for each variable, t is the biomineralization time, and R corresponds to the salt/protein ratio.
Coefficients for both models were determined by the least square regression method using the *solver* tool available in Microsoft Excel.

Table S6. Parameters of the models developed for the immobilization yield (IY) and specific activity (SA) of the biomineralization of β-galactosidase with calcium-phosphate according to Equation S4.

	β_0	β_1	β_2	β_{12}	β_{11}	β_{22}
IY [%]	-60.00	0.15	0.64	$-2.47\cdot10^{-4}$	$-6.82\cdot10^{-4}$	$-1.03\cdot10^{-3}$
SA [IU/g] x10^3	-9.90	0.06	0.23	$-1.19\cdot10^{-4}$	$-2.70\cdot10^{-4}$	$-4.38\cdot10^{-4}$

Regression models for the experimental designs

Results from the experimental run at pH 9.0 were modeled according to a multiple linear regression as represented by the following expression:

$$y = \beta_0 + \beta_1 R + \beta_{11} R^2 \qquad \text{Equation S2}$$

Where y could be SA or IY, β_0, β_1 and β_{11} are the model coefficients for each variable, t is the biomineralization time, and R corresponds to the salt/protein ratio.
Coefficients for both models were determined by the least square regression method using the *solver* tool available in Microsoft Excel.

Table S7. Parameters, p-values, and coefficients of determination (R^2) of the models developed for the immobilization yield (IY) and specific activity (SA) resulting from the biomineralization of β-galactosidase at pH 9.0 for 60 min. The models are represented by Equation S5.

	β_0	β_1	β_{11}	p-value	R^2
IY [%]	-58.16	0.85	$-1.3\cdot10^{-3}$	$1.6\cdot10^{-4}$	0.982
SA [IU/g] x10^3	6237.19	227.71	-0.46	$4.6\cdot10^{-4}$	0.972

The resulting kinetics were modeled after a series biphasic inactivation mechanism without residual activity [37]. According to such mechanism, inactivation proceeds in two succeeding stages in which an intermediate state of non-zero activity transitions to a final condition of null residual activity. The mechanism is represented by Equation S3:

$$\frac{A(t)}{A_0} = \left[\left[1 + \alpha \cdot \left[\frac{k_1}{k_2 - k_1}\right]\right] \cdot \exp\left(-k_1 \cdot t\right) - \alpha \cdot \left[\frac{k_1}{k_2 - k_1}\right] \cdot \exp\left(-k_2 \cdot t\right)\right]$$

(Equation S3)

Where $A(t)$ and A_0 represent the activity at any time t and the initial activity respectively (IU/g); k_1 and k_2 are the inactivation rate constants of the first and second inactivation stages (h^{-1}); and α is the specific activity ratio between the activity of the intermediate state and A_0.

The model coefficients were determined by the least square nonlinear regression method. Half-life time ($t_{1/2}$, h), which is the time at which the enzyme has lost 50% of its initial activity, and stabilization factor (SF) were calculated for each inactivation kinetics. These parameters are defined according to the following equations:

$$t_{1/2} = t_{E=E_0/2}$$ Equation S4

$$SF = \frac{t_{1/2\ biocatalyst}}{t_{1/2\ free\ enzyme}}$$ Equation S5

Where $t_{E=Eo/2}$ is the time at which activity reaches half of the initial activity.
Another parameter used to assess the performance of the immobilized biocatalyst is the catalytic potential (CP) which allows to combine stability and specific activity into one compounded parameter [37]. CP denotes the total catalytic capacity of the biocatalyst along its lifespan, and is defined by Equation S:

$$CP = \int_{t=0}^{t=t_r} A(t) \cdot dt$$ Equation S6

Where A(t) denotes the activity of the biocatalyst as a function of time as represented by the inactivation model (Equation S3), and tr is the time corresponding to the replacement criterion defined as the time when the catalyst should be discarded. In this case, a replacement at 30% residual activity was considered.

Table S8. Model parameters, *p*-values, and coefficients of determination of the kinetic models of inactivation of β-galactosidase nanocrystals produced at pH 7.4 and pH 9.0 for 60 min of biomineralization at calcium/protein ratios of 115 $mmol_{CaCl2}/g_{protein}$ (R1), 290 $mmol_{CaCl2}/g_{protein}$ (R3), and 460 $mmol_{CaCl2}/g_{protein}$ (R6). The models correspond to the biphasic inactivation mechanism as represented by Equation S6.

	**k_1 [h^{-1}]**	**k_2 [h^{-1}]**	α	***p*-value**	**R^2**	**SF**
β-gal	0.157	0.027	0.259	1.5×10^{-10}	0.998	1.0
pH7.4 _R1	0.074	0.013	0.473	1.7×10^{-6}	0.995	3.4
pH7.4_R3	0.057	0.010	0.468	8.2×10^{-8}	0.992	4.3
pH7.4_R6	0.092	0.011	0.639	3.4×10^{-11}	0.999	5.3
pH9.0_R1	0.042	0.007	0.634	3.4×10^{-7}	0.988	9.4
pH9.0_R3	0.214	0.007	0.866	8.5×10^{-8}	0.991	11.6
pH9.0_R6	0.328	0.006	0.903	2.2×10^{-7}	0.989	14.7
